# Mortality among Cancer Patients within 90 Days of Therapy in a Tertiary Hospital, Tanzania: Is Our Pretherapy Screening Effective?

**DOI:** 10.1155/2020/4274682

**Published:** 2020-08-12

**Authors:** Marygoreth J. Changalucha, Martha F. Mushi, Rodrick Kabangila, Vitus Silago, Beda Likonda, Stephen E. Mshana

**Affiliations:** ^1^Department of Internal Medicine, Bugando Medical Centre/Catholic University of Health and Allied Sciences, Mwanza, Tanzania; ^2^Department of Microbiology and Immunology, Catholic University of Health and Allied Sciences, Mwanza, Tanzania; ^3^Kamanga Medics Hospital, Mwanza, Tanzania; ^4^Oncology Department, Bugando Medical Centre/Catholic University of Health and Allied Sciences, Mwanza, Tanzania

## Abstract

**Background:**

A high mortality has been reported during the first ninety days of cancer therapy and is more pronounced in patients with febrile neutropenia. The Bugando Medical Center oncology department offers cancer diagnosis and treatment services to the population of the Lake Zone of Tanzania with limited data on the outcome within 90 days of therapy. Here, we report the 90-day mortality and factors associated with it among cancer patients attending the oncology department of the tertiary hospital in Tanzania. *Methodology*. Enrolled participants underwent baseline physical examinations, and their functional status was assessed using Karnofsky score. On each clinic visit, full blood picture was taken and patients were investigated for infections. Data were entered in the Microsoft Excel, cleaned and coded and then transferred to STATA version 13 for analysis.

**Results:**

A total of 102 participants were included in the final analysis. Their median age was 50 years (38-60). The majority of the study participants were females 76 (75%), and 82 (80.4%) had primary school education. The majority of the patients had solid cancer 96 (94.1%). A total of 12 (11.8%) patients died within 90 days of starting therapy. Low hemoglobin level at the start of cancer therapy, Karnofsky score below 80%, and using 5-fluorouracil-containing therapy were statistically significantly found to be associated with mortality within 90 days of therapy among cancer patients.

**Conclusion:**

One tenth of cancer patients at Bugando Medical Center do not survive within 90 days of therapy; the mortality is significantly high among anemic patients, with poor performance status, on 5-fluorouracil regimen, and diagnosed with head and neck cancer, necessitating close follow-up of these patients.

## 1. Background

Cancer is the second leading cause of death worldwide [1], with more than 60% of cases being reported from Africa, Asia, and Central and South America. These regions account for about 70% of cancer deaths worldwide [2]. It was estimated that between 2000 and 2020 there would be 75% increase of cancer incidence in resource-limited countries [3]. Mortality rate due to cancer is more in resource-limited countries than in developed countries [4]. It is estimated that by 2020, about 70% of deaths associated with cancer diseases would be from resource-limited countries and cancer survival rate would be 50% less than that of the developed countries [5].

In Tanzania, by the year 2008, there were 21,180 new cases of cancer per 42.5 million people [6]. In 2014, deaths due to cancers were reported to be 9,100 and 10,800 for men and women, respectively [7]. Cervical and breast cancers were leading causes of death among women while esophagus and head and neck cancers were leading causes of death among men [7]. Mortality among cancer patients is reported to be significantly high within the first 90 days of therapy [8, 9], and the incidence is reported to be 10.3 times higher in patients with one comorbidity (renal disease, heart disease, chronic bronchitis, or mucositis) than those without comorbidity and can increase to 24.1 times higher with the increase in number of comorbidities [8]. Other factors associated with mortality among cancer patients on therapy include the type of cancer, anticancer regimen, age, performance status, anemia, and neutropenia [8, 10, 11]. Furthermore, outpatients have been reported to have higher mortality rate than the inpatients which is associated with the lack of supportive care among outpatients [8, 10].

The number of cancer patients receiving medication at the oncology department of Bugando Medical Center (BMC) has increased due to the availability of chemotherapy, diagnostic techniques, and improved infrastructure for cancer management. However, data on mortality and its associated factors within 90 days of starting chemotherapy is still limited. Here, we are reporting mortality and factors associated with it within 90 days of therapy among cancer patients attending the oncology department of BMC. The spectrum of bacterial pathogens causing infections among these patients has also been determined.

## 2. Methods

This was a hospital-based cross-sectional study with a follow-up component which was conducted from August 2017 to May 2018 at the BMC oncology department. The unit attends about 100-150 cancer patients per month and receives up to 25 new patients per month (http://www.bugandomedicalcentre.go.tz/index.php?bmc=24). The study enrolled all patients with age above 12 years, histologically confirmed to have cancer and scheduled to start chemotherapy.

The minimum number of patients to be enrolled in the study was obtained using Kish and Lisle formula of 1965 [12]. The prevalence of mortality among cancer patients of 8% was substituted, and the minimum number of patients obtained was 82 [9].

### 2.1. Data Collection

A total of 114 patients were eligible to start cancer therapy at the BMC oncology department during the study period; however, we enrolled 111 patients because 3 patients declined participation.

At enrollment, physical and clinical examination was done; blood pressure was measured using a digital blood pressure monitor CHU 304 (Citizen Systems Japan Co. Ltd.) that has been clinically validated by the European Society of Hypertension protocol of 2010. Body temperature was measured by a digital thermometer MDD 93/42/EEC, “0197” (Holding Corp. GmbH (Hamburg)), and body weight and height were measured using DETECTO machine (Webb City, MO, USA). The data on type, stage of cancer, treatment regimen, and duration of therapy were recorded from the patients' file. At each visit, patients were evaluated for signs and symptoms of infections, especially fever.

At baseline, the following investigations were done: full blood picture (FBP), and urine and blood culture as per the BMC management protocol. Another FBP was taken during follow-up visits, and for patients who developed signs and symptoms of infections, blood and urine were collected for culture following the standard operating procedures of the accredited microbiology laboratory of BMC. Blood cell counts were analyzed by the use of Cell-Dyn 1800 automated hematology analyzer (Abbott Diagnostics, Illinois, USA). Drug susceptibility testing was done using Kirby Bauer disk diffusion technique and interpreted using the Clinical and Laboratory Standards Institute (CLSI) guidelines [13].

### 2.2. Data Management and Analysis

Laboratory data were recorded in a laboratory workbook and then entered onto the Excel spreadsheet for cleaning and coding before being transferred to STATA version 13 for analysis. Continuous data like blood pressure, body weight, Karnofsky score, temperature, white blood cell count, and neutrophil count were presented as medians and interquartile ranges and compared by the Wilcoxon rank-sum test. Categorical variables such as sex, marital status, occupation, and level of education were presented as frequency (percent) and compared by chi-square or Fisher's exact test. A *p* value of <0.05 was considered statistically significant.

## 3. Ethics Approval and Consent to Participate

The protocol for this study was ethically approved by the joint CUHAS/BMC ethics and scientific review committee with certificate number CREC/228/2017. Permission to carry out the study was also sought from the management of the BMC and the oncology department. Written informed consent was obtained from the participants after being informed about the study objectives and procedures. For all participants aged less than 18 years, consents were sought from parents/guardians and assent was requested from the participant.

## 4. Results

### 4.1. Social Demographic and Baseline Clinical Data

Out of 111 enrolled patients, 102 were included in the final analysis. A total of 5 patients could not start cancer therapy due to financial reasons; two were lost to follow-up, one died before therapy was initiated and one shifted to a referral cancer hospital (Ocean Road Cancer Institute, Dar es Salaam, Tanzania).

The median age (IQR) of the study population was 50 (30-60) years. The majority were females (76, 74.5%), and (82 80.3%) had primary school education. The majority (70, 68.6%) were married. The majority of patients had solid cancers (94.1%), and about one-third of patients reported at the oncology department while at cancer stage IV. All patients in stage IV of cancer received palliative care and low-dose chemotherapy calculated based on their performance status. Regarding various clinical parameters, the median body temperature (IQR) was 36.2°C (35.6-36.6), with median neutrophil (IQR) count of 3.0 × 10^9^/L (2.1-4.1) and median Hb 11.6 g/dL (10.2-12.6). Out of 102 patients, 19 (18.6%) were HIV positive.

### 4.2. Types of Cancer Diagnosed

The commonest type of cancer diagnosed was gynecological cancer (47, 46.1%) which was led by cervical cancer (40, 39%), followed by head and neck cancer (12, 11.8%) led by squamous cell carcinoma of the head and neck (6, 4.9%). Breast cancer was diagnosed in 11 (10.8%) (Table 1).

### 4.3. Mortality within 90 Days of Starting Chemotherapy

Mortality within 90 days of starting therapy among cancer patients at the oncology department was 12 (11.8%). The age of those who died ranged between 33 and 76 years. All patients who died in the current study had solid cancer and anemia at baseline with hemoglobin range from 7.3 to 11.4 g/dL. The majority (11, 91%) reported at the oncology department at advanced stage, i.e., cancer stages 3 and 4. A total of 9 (75%) had a performance status below 80% by Karnofsky score (Table 2).

### 4.4. Culture Results and Mortality within 90 Days of Starting Chemotherapy

Among the 102 nonrepetitive blood cultures done at baseline, 7 (6.9%) were culture positive. *S. aureus* (4, 3.9%) was the most frequently detected pathogen, followed by *E. coli* and *Pseudomonas aeruginosa*, 2 (1.9%) and 1 (1%), respectively. Regarding urine culture, 13/102 (12.7%) patients had significant bacteriuria at baseline. *S. aureus* (7, 6.9%) was the most frequently isolated bacteria species, followed by *E. coli* (5, 4.9%) and *Pseudomonas aeruginosa* (1, 1%). Of the 18 patients with bacterial infections, either UTI or blood stream infections, 3/18 (16.6%) died compared to 9/84 (10.8%) without bacterial infection, *p* = 0.244. Of the patients with bacterial infections who died, one had *S. aureus* blood stream infection and two had significant bacteriuria, one due to *P. aeruginosa* and another due to *E. coli*.

### 4.5. Factors Associated with Mortality

Significant proportion of patients with head and neck cancer died compared to patients with other types of cancer (33.3% (4/12) vs. 8.9% (8/90), *p* = 0.014). The median hemoglobin level of patients who died was significantly lower than the median hemoglobin level of patients who survived (10 (9.3-10.8) g/dl vs. 11.8 (10.5-12.7) g/dl, *p* = 0.001) (Table 3).

## 5. Discussion

Mortality within 90 days of starting chemotherapy among cancer patients in the current study was found to be 12%. The mortality reported was almost similar to 11% that was reported by Kuderer et al. from the United States among cancer patients on therapy in 2006 and slightly higher than 8% which has recently been reported from South Africa [8, 14]. Furthermore, the mortality was significantly high compared to 7.2% reported by the International Agency for Research on Cancer from Sub-Saharan Africa in the global cancer statistics of 2012 [15]. Additionally, similar mortality was reported in the study done in West Africa 8 years ago which reported the mortality of 10% [16]. Low functional status due to cancer itself and the toxicity brought by the therapy collectively compromise the health of the patients and might lead to high mortality observed after therapy initiation in the current study.

As previously reported in the world cancer report of 2012 [17], the current study found solid cancers to be the predominant cancer type at BMC. However, contrary to the world report which observed the commonest solid cancer to be lung cancer [18, 19], this study observed cervical cancer to be the commonest solid cancer. In Tanzania, cervical cancer has been the leading cancer in the past one decade [20, 21]. The ongoing national campaign regarding screening for cervical cancer could explain the findings. Furthermore, it should be noted that maybe lung cancer is underreported due to the little attention that is given to it.

Contrary to high mortality that has been reported in several studies [8, 22] among patients with hematological malignancies, in the current study, all patients with hematological malignancy survived within 90 days of therapy. Hematological malignancy is known to have higher mortality than solid cancer, the reason being that hematological malignancies are associated with both depletion and impaired function of neutrophils due to cancer itself and the toxicity of therapy [22, 23]. The mortality data for patients with hematological malignancy in the current study should be carefully interpreted due to the limited number of patients.

In the current study, severe neutropenia was not observed. Neutropenia has been associated with increased mortality in many studies [22, 23]. In the current study, the use of granulocyte colony-stimulating factors among patients with reduced number of neutrophil before starting therapy prevented severe neutropenia hence reduced mortality of these patients.

As in a previous study in the United States and Greece, significantly more mortality was observed among patients in 5-fluorouracil treatment [24]. The toxicity induced by 5-fluorouracil such as stomatitis, mucositis, and diarrhea [24] could partly explain the observation. As observed in a previous study in France [25], patients with carcinoma of the head and neck had two times odds of dying than patients with other cancers.

This study observed increased mortality in head and neck cancer patients; this could be due to the fact that the disease is close to vital organs with possibility of impairment in swallowing and breathing [26]. Additionally, significantly high mortality among patients with head and neck cancers in this study could be contributed by late stage presentation at the oncology department, advanced age, and poor performance status. Late stage presentation has been documented as the main predictor of mortality [26]. Furthermore, advanced age has been noted as the poor prognostic factor among patients with head and neck cancer [27].

As it was documented in a meta-analysis where anemia was found to be the leading cause of mortality [28], in the current study, all patients who died had anemia at the baseline. This could be due to the fact that anemia leads to the hypoxic state which reduces the amount of drug delivered to the cell and hence reduces the therapy response [29, 30]. The findings of this study regarding high mortality among anemic patients confirm what has been reported in previous studies [28, 31]. Furthermore, anemia is associated with fatigue which causes the inability to perform daily activities among cancer patients and hence reduces the quality of life of patients and increases mortality [29, 30].

## 6. Conclusion

The mortality of cancer patients within 90 days of therapy was slightly higher than that of patients in Sub-Saharan Africa and more pronounced in patients with anemia at baseline, use of 5-fluorouracil, poor performance status, and diagnosis of head and neck cancers necessitating close follow-up of these patients. Furthermore, a study focused on hematological cancers from developing countries to provide data on mortality is recommended.

## Figures and Tables

**Table 1:** Description of type of cancer seen during the study period.

Variable	Frequency (*N*)	Percent (%)
*Gynecological cancers*	**47**	**46**
Cancer of the cervix	40	39
Uterine cancer	5	4.9
Ovarian cancer	1	1
Choriocarcinoma	1	1
*Head and neck cancers*	**12**	**12**
Squamous cell carcinoma of the head and neck	5	4.9
Adenocarcinoma of the head and neck	3	2.9
Retinoblastoma	2	2
Nasopharyngeal cancer	1	1
Sinonasal cancer	1	1
*Connective tissue cancers (sarcoma)*	**12**	**12**
Kaposi's sarcoma	6	6
Malignant melanoma	2	2
Malignant phyllodes	1	1
Fibrosarcoma	1	1
Osteosarcoma	1	1
Chondrosarcoma	1	1
*Breast cancer*	**11**	**11**
*Gastrointestinal cancers*	**10**	**10**
Hepatocellular cancer	4	4
Pancreatic cancer	2	2
Colon cancer	2	2
Esophageal cancer	1	1
Biliary rhabdomyosarcoma	1	1
*Hematolymphoid malignancies*	**6**	**6**
Hodgkin lymphoma	2	2
Chronic myeloid leukemia	1	1
Acute myeloid leukemia	1	1
Burkitt's lymphoma	1	1
Non-Hodgkin lymphoma	1	1
*Genital and reproductive cancer*	**2**	**2**
Perianal cancer	1	1
Prostate cancer	1	1
*Lung cancer*	**1**	**1**

**Table 2:** Baseline characteristics for patients who died within 90 days.

Variable	Patient identification											
	1	2	3	4	5	6	7	8	9	10	11	12
Age (years)	76	71	52	43	65	50	49	62	51	51	52	33
BMI (kg/m^2^)	18	29	22.2	25	21	25	27.7	23.2	25.2	22.8	24.8	20.8
Cancer type	Nasopharyngeal cancer	Breast cancer	GIT metastatic cancer	Malignant melanoma	Cervical cancer	Pancreatic cancer	Sinonasal cancer	Adenocarcinoma of the nose	Cervical cancer	Cervical cancer	Lung cancer	Squamous cell carcinoma of the cheek
Type of therapy												
Type 1 Type 2 Type 3	5-Fluorouracil Cisplatin Paclitaxel	Carboplatin	Docetaxel Dexamethasone	Carboplatin	Cisplatin Dacarbazine	Gemcitabine 5-Fluorouracil	Cisplatin Radiotherapy	Cisplatin	Cisplatin Radiotherapy	Cisplatin 5-Fluorouracil	Docetaxel Dexamethasone	Cisplatin 5-Fluorouracil Dexamethasone
Stage of cancer	3	4	4	3	3	3	4	4	2	3	3	3
Baseline neutrophil (×10^9^/L)	3.51	19.7	3.2	2.38	2.94	5.65	0.75	2.83	5.79	3.21	3.05	1.47
Neutrophil 1 (×10^9^/L)	2.9	19.8	2.87	3.92	1.09	5.17	1.65	2.6	5.8	2.25	2.4	1.2
Neutrophil 2 (×10^9^/L)	—	—	1.35	2.41	1.08	—	1.41	1.6	7.46	—	1.3	1.7
Neutrophil 3 (×10^9^/L)	—	—	—	1.13	—	—	—	3.17	2.69	—	—	—
Neutrophil 4 (×10^9^/L)	—	—	—	—	—	—	—	3.07	3.91	—	—	—
Neutrophil 5 (×10^9^/L)	—	—	—	—	—	—	—	1	—	—	—	—
Baseline Hb (g/dL)	9.6	11.1	11	9.9	10.1	9	9.1	10.5	7.3	10.3	11.4	9.5
Baseline WBC (×10^9^/L)	6.14	4.58	4.8	4.29	5.33	8.37	2.92	4.8	8.84	5.1	4.97	2.29
Karnofsky score (%)	80	20	60	80	90	70	90	70	80	90	80	70
Blood culture	No growth	No growth	No growth	No growth	No growth	No growth	No growth	No growth	No growth	No growth	*S. aureus*	No growth
Urine culture	No growth	*P. aeruginosa*	No growth	No growth	No growth	No growth	No growth	No growth	No growth	No growth	No growth	*E. coli*
Body temperature(°C)	36	38.2	37.1	36.3	36.8	37.2	37	35.2	35.5	36.7	35.3	36.8

**Table 3:** Factors associated with mortality within 90 days of therapy.

Variable	Dead (12)	Alive (90)	*p* value
	Number (%) median [IQR]	Number (%) median [IQR]	
HIV status			
Unknown (21)	3 (14.3)	18 (85.7)	
Negative (62)	9 (14.5)	53 (85.5)	
Positive (19)	0 (0)	19 (100)	0.211
Baseline body temperature (°C)	36.2 [35.4-36.4]	36.2 [35.7-36.7]	0.4023
BMI	23.8 [21.7-25]	22.9 [20.2-25.9]	0.453
Cancer type			
Others (90)	8 (8.9)	82 (91.1)	
Head and neck (12)	4 (33.3)	8 (66.67)	0.014
Therapy type			
Cisplatin (50)	7 (14.0)	43 (86.0)	0.492
5-Fluorouracil (10)	4 (40.0)	6 (60.0)	0.004
Paclitaxel (22)	1 (4.5)	21 (95.5)	0.235
Carboplatin (9)	2 (22.2)	7 (77.8)	0.308
Dacarbazine (4)	1 (25.0)	3 (75)	0.402
Docetaxel (6)	2 (33.3)	4 (66.7)	0.091
Karnofsky score (%)	80 [70-85]	90 [80-90]	0.001
Baseline Hb (g/dL)	10 [9.3-10.8]	11.8 [10.5-12.7]	0.001
Baseline white blood cell (×10^9^/L)	4.9 [4.4-5.7]	5.2 [4-6.7]	0.226
Baseline neutrophil (×10^9^/L)	3.1 [2.6-4.6]	3 [2-4.1]	0.653
Body temperature visit (°C)			
Visit 1	36.6 [36.1-37]	36.4 [35.9-36.8]	0.852
Visit 2	36.7 [36-36.9]	36.2 [35.8-36.8]	0.677
Visit 3	36.1 [36.1-36.3]	36.3 [35.8-36.8]	0.565
Visit 4	36.3 [35.5-37.1]	36.2 [35.7-36.6]	0.758
Visit 5	35.2 [35.2-35.2]	36.4 [35.9-37]	0.490

## Data Availability

The data is available upon request and the request should be made to the Director of Research and Innovation, Catholic University of Health and Allied Sciences.

## Abbreviations

BMC: Bugando Medical Centre
CLSI: Clinical and Laboratory Standards Institute
FBP: Full blood picture
IQR: Interquartile range.
